# Measurement properties of instruments assessing the clinical learning environment in health professions education: a COSMIN-based systematic review

**DOI:** 10.3352/jeehp.2026.23.14

**Published:** 2026-06-11

**Authors:** Matthias Michael Walter, Alexander P Schurz, Nele Adriaenssens, Evert Zinzen, Slavko Rogan

**Affiliations:** 1 Department of Movement and Nutrition for Health and Performance, Faculty of Physical Education and Physiotherapy, Vrije Universiteit Brussel, Brussels, Belgium; 2 Science and Research, Physio Insight, Haslach im Kinzigtal, Baden-Württemberg, Germany; 3 Department of Health Professions, Bern University of Applied Sciences, Bern, Switzerland; 4 Rehabilitation Research Group, Faculty of Physical Education and Physiotherapy, Vrije Universiteit Brussel, Brussels, Belgium; 5 Medical Oncology Department, Universitair Ziekenhuis Brussel, Brussels, Belgium; The Catholic University of Korea, Korea

**Keywords:** Clinical competence, Health occupations, Patient-reported outcome measures, Professional education, Students

## Abstract

**Purpose:**

The clinical learning environment (CLE) is a crucial component of health professions education, providing the foundation for developing profession-specific clinical skills. This systematic review aimed to identify evaluated assessment tools for the CLE in health professions education and to report their measurement properties.

**Methods:**

This systematic review was preregistered (IDESR000098), and its protocol was published previously. Eligible studies were peer-reviewed articles in English that developed and validated tools for assessing the CLE among undergraduate health professions students. The review followed the COSMIN guidelines for systematic reviews of patient-reported outcome measures. Multiple electronic databases, including MEDLINE, the Cochrane Library, ERIC, Education Research Complete, and CINAHL, were searched; studies were independently screened, and data were extracted. Data were synthesized using best-evidence synthesis according to COSMIN guidelines.

**Results:**

Of the 6,236 records screened by title and abstract, 55 were eligible for full-text screening. A supplementary search identified 13 additional articles, resulting in 39 included articles. Overall, 28 tools were identified, with 4 tools (PET, PET-Midwifery, DECLEI, and MidSTEP) demonstrating sufficient content validity. Only MidSTEP demonstrated sufficient structural validity.

**Conclusion:**

Only a minority of the included tools provided sufficient evidence of content and structural validity according to the COSMIN criteria. This finding indicates a systemic need for higher standards in monitoring clinical placements and identifies tools that should be re-evaluated and supported by additional research. Limitations include the exclusion of EMBASE and gray literature and the reliance on studies that predominantly used psychometric-first rather than content-validity-first designs.

## Graphical abstract

[Fig f2-jeehp-23-14]

## Introduction

### Background

In health professions education, clinical education (CE) plays a crucial role. It consists of practical teaching at universities or higher education institutions (HEIs) and internships in which students encounter authentic clinical practice. During these internships, the clinical learning environment (CLE) represents a complex social context in which students apply clinical skills in real-life practice settings while ensuring patient safety and observing potential role models. Research has shown that a positive CLE supports students’ competence development [1]. Therefore, systematic evaluation of the CLE is crucial for ensuring the competence development of future health professionals.

Discourse-analytic work in higher and health professions education shows that quality is not a neutral concept but is shaped by different institutional logics, such as externally driven quality control, efficiency-oriented managerial approaches, and participatory and culturally oriented perspectives on organizational learning [2]. These discourses influence which aspects of the CLE are captured in assessment instruments and which dimensions, particularly cultural or relational aspects, may be underrepresented. Against this background, a systematic review can not only identify evaluated instruments but also highlight conceptual gaps in CLE measurement and inform more targeted future instrument development.

Given the importance of the CLE, previous research has identified various tools used to assess the CLE across health professions [3,4]. However, existing reviews have important methodological limitations. Search strategies often failed to capture all relevant concepts and were not conducted in accordance with COSMIN guidelines for measurement instrument evaluation [3,4].

To date, no systematic review has identified evaluated instruments for assessing the CLE in undergraduate education across different health professions. Including multiple professions and applying COSMIN guidelines therefore allows for a comprehensive overview of evaluated CLE assessment instruments.

### Objectives

This systematic review aimed to identify and evaluate instruments for assessing the CLE in undergraduate health professions education and to report their measurement properties. By applying the COSMIN guidelines, version 2.0 [5], this review sought to identify existing gaps and highlight opportunities to advance the measurement and improvement of CLE quality across different health disciplines.

## Methods

### Ethics statement

This was a literature-based study. Therefore, neither institutional review board approval nor informed consent was required.

### Study design

This systematic review was preregistered in the International Database of Education Systematic Reviews under registration number IDESR000098, and its protocol was published previously [6]. Any significant updates or changes to the published protocol are documented below in the subsection on protocol deviations. This study followed the Consensus-Based Standards for the Selection of Health Measurement Instruments (COSMIN) methodology for conducting systematic reviews of patient-reported outcome measures (PROMs), version 2.0 [5], and the Preferred Reporting Items for Systematic Reviews and Meta-Analyses (PRISMA) guidelines for reporting the research process [7].

### Protocol deviations

Compared with the registered protocol, EMBASE and the Education Database were not included in the final search. EMBASE was excluded due to the low specificity of the search results, and the Education Database was excluded due to lack of access. No other significant deviations from the protocol were identified.

### Eligibility criteria

We considered peer-reviewed articles in English in which self-reported questionnaires completed by undergraduate health professions students were developed and evaluated to assess the quality of the CLE in higher education in the health professions. No publication date limit was defined. Articles focusing on other aspects of CE, such as postgraduate internships or medical residency programs; tools targeting evaluations by clinical supervisors or learning environments at HEIs or universities; study protocols; abstracts; conference papers; and dissertations were excluded. Gray literature was excluded to ensure methodological rigor and to focus exclusively on peer-reviewed studies, which are typically subject to a higher standard of quality assurance.

### Information sources

MEDLINE (accessed via Ovid), the Cochrane Library, ERIC, Education Research Complete, and CINAHL (accessed via EBSCO) were searched for relevant articles.

### Search strategy

The primary database search was conducted in May 2025, followed by a structured supplementary search in December 2025 to ensure the identification of recently published studies and a comprehensive evaluation of the measurement properties of the identified instruments. The SPIDER framework (Sample, Phenomenon of Interest, Design, Evaluation, and Research Type) was applied to structure the search strategy and support comprehensive concept mapping rather than to restrict study designs.

A comprehensive search strategy, developed in collaboration with an experienced librarian, was implemented to identify relevant studies. First, a search strategy for MEDLINE was developed and subsequently adapted for the other databases. The search strategies included keywords as well as Medical Subject Headings (MeSH terms), depending on the database, and were supplemented with synonyms. We intentionally excluded specific research types, such as “mixed methods,” “qualitative,” and “quantitative,” from the search strategy to avoid unintentionally omitting potentially relevant articles focusing on assessment tools and their measurement properties. Keywords and index terms were tailored to each information source and combined using Boolean operators.

In addition to the primary database search, a structured supplementary search was conducted to ensure a comprehensive evaluation of the measurement properties of instruments identified for inclusion and to capture additional relevant instruments that may not have been retrieved in the primary search because of inconsistent indexing or very recent publication. For each included instrument, we performed (1) backward citation searching by screening the reference lists of the original studies, (2) forward citation searching using citation-tracking functions in Google Scholar to identify additional development or validation studies related to the same instrument, and (3) tool-specific searches in PubMed using a standardized search string pattern. The full reproducible search strategies for all databases are provided in the supplementary material (Supplement 1. Search strategy).

### Selection process

Two reviewers (M.W. and A.S.) independently screened titles and abstracts against the eligibility criteria. Any articles deemed relevant by at least 1 reviewer were included in the full-text review. If the relevance of a study was unclear from the abstract, the full article was independently assessed by the 2 researchers (M.W. and A.S.). To gauge inter-rater agreement, Cohen’s κ statistic was computed for the initial title and abstract screening and full-text screening. Any discrepancies during the screening process were resolved through discussion with a third reviewer (S.R.). Rayyan was used for the screening process.

### Data collection process

Data extraction was conducted as recommended in the COSMIN guidelines [5]. One reviewer (M.W.) extracted data from the included articles, and a second reviewer (A.S.) independently checked the extracted data. A modified version of the extraction table provided by COSMIN was used. In cases of disagreement, a third reviewer (S.R.) was involved to reach consensus. Where required, authors were contacted to request missing or additional data. The data were entered into an Excel spreadsheet provided by COSMIN, which was pilot-tested on 5 studies.

### Content validity assessment

Content validity is considered the most essential measurement property and a prerequisite for meaningful interpretation of other psychometric characteristics [5]. Therefore, content validity was assessed first in accordance with COSMIN recommendations, using Box 1 and Box 2 of the COSMIN Risk of Bias checklist and the 10 COSMIN content validity criteria, version 2.0 [5]. If the tool was not published with the article, it was excluded from further assessment of measurement properties, as recommended by the COSMIN guidelines [5].

Involvement of the target population was required to establish relevance, comprehensiveness, and comprehensibility, as these aspects cannot be confirmed by expert judgment alone. Reviewer ratings were not used as a substitute when such involvement was lacking or unclear.

### Study-level and tool-level decision rules

If the target population was not involved, content validity was rated as indeterminate at the study level because relevance and comprehensibility can only be evaluated by the target population [5]. Study-level results were also rated as indeterminate when methodological quality was rated as inadequate in Box 1 or Box 2, as results from such studies were considered uninterpretable. Following COSMIN guidance, indeterminate results at the study level cannot lead to a sufficient content validity rating at the tool level. Consequently, self-reported questionnaires for which no evidence involving the target population was available, or for which all available studies were rated as indeterminate, were rated as having insufficient content validity at the tool level.

### Eligibility threshold for further psychometric synthesis

Given the large number of available self-reported questionnaires in this domain and to avoid misleading precision for instruments without demonstrated content validity, a conservative eligibility threshold was applied. Only tools with sufficient (+) content validity were retained for subsequent synthesis of measurement properties and recommendations. Instruments rated as insufficient (−), inconsistent (±), or indeterminate (?) at the tool level were reported for transparency but were not subjected to further synthesis.

Internal consistency and reliability can be interpreted only when the dimensionality of the instrument is adequately supported [5]. When structural validity is insufficient, these properties are considered indeterminate regardless of the reported coefficients.

### Use of existing COSMIN ratings

Content validity ratings from prior systematic reviews were adopted when available [5]. For instruments covered by previous COSMIN-based reviews, these ratings were retained to avoid duplication of assessments and to ensure consistency. When prior COSMIN ratings were based on earlier criteria or did not fully assess content validity according to current standards, such as lack of target population involvement, instruments were re-evaluated. Instruments not evaluated in prior reviews were assessed de novo following the COSMIN framework.

### Data items

Basic characteristics of each identified tool were listed, including the name, authors, publication year, targeted health profession, number of factors and items, and reported psychometric properties.

### Risk of bias assessment

The risk of bias of the included studies was assessed by 2 reviewers (M.W. and A.S.) using the COSMIN Risk of Bias checklist for systematic reviews of PROMs, version 3.0 [5]. A consensus meeting was organized with a third researcher (S.R.) in cases of disagreement.

### Effect measures

The primary aim of this systematic review was to identify evaluated assessment tools for measuring the quality of the CLE in undergraduate health professions education and to report their measurement properties according to the COSMIN guidelines. Depending on the property assessed, reliability coefficients, such as Cronbach’s alpha, intraclass correlation coefficient, and person separation index (PSI), and structural validity indices, such as factor loadings and fit indices including comparative fit index, Tucker-Lewis index, root mean square error of approximation, and standardized root mean square residual, were reported. Each index of the assessment tools that provided sufficient content validity was interpreted according to the COSMIN criteria for good measurement properties. Evidence was synthesized per instrument and per measurement property using a best-evidence synthesis.

### Synthesis of results

The primary unit of analysis in this review was the instrument. When multiple studies were available for a given instrument, evidence was synthesized across studies in accordance with COSMIN guidelines. Basic characteristics of each identified tool were listed, including the name, authors, publication year, targeted health profession, number of factors and items, and reported psychometric properties. Tools providing sufficient content validity are presented in a separate table from tools with insufficient content validity. The results of the risk of bias assessment of the tools with sufficient content validity were summarized for each tool in a separate table.

### Reporting bias assessment

Reporting bias was assessed qualitatively by 2 reviewers (M.W. and A.S.) by comparing the measurement properties described in the methods sections of the included studies with those reported in the results. Reporting of measurement indices was noted when analyses were described but results were absent.

### Certainty assessment

The certainty of evidence for each measurement property was evaluated using the COSMIN-adapted GRADE (Grading of Recommendations Assessment, Development and Evaluation) approach [5]. Two reviewers (M.W. and A.S.) independently rated the certainty of evidence, with disagreements resolved by consensus with a third reviewer (S.R.). Results were integrated into an instrument-level summary table that combined measurement property ratings and evidence certainty to support interpretation and comparison across tools.

## Results

### Study selection

A total of 8,551 records were identified through database searches. After removal of 2,315 duplicates, 6,236 records were screened by title and abstract. Fifty-five articles were eligible for full-text screening and were sought for retrieval, of which 6 could not be retrieved. Inter-rater agreement was excellent for title and abstract screening (κ=0.98) and full-text screening (κ=0.89).

Additional searches identified 13 articles, including 5 articles describing additional instruments [8-12] and 8 supporting validation or development studies of already included instruments [13-20]. Of the 62 records assessed at full text, 23 were excluded for the following reasons: adaptation study (n=1); different population (n=10); wrong outcome (n=10); and wrong study design (n=2).

**Fig. 1.**
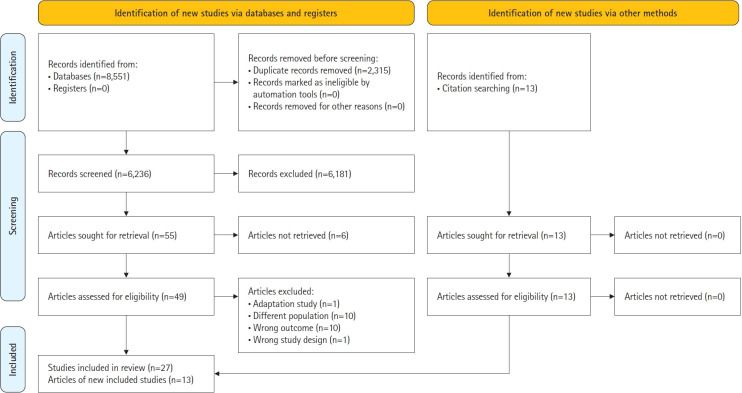
PRISMA flow chart.

In total, 39 articles describing 28 distinct instruments were included in the review [8-46]. The PRISMA flow chart is shown in Fig. 1.

### Study characteristics

The largest group of tools was designed for nursing (*k*=14 instruments, n=22 studies) [8,12,14-19,23-26,29-31,34,39,41,43-46], followed by medicine (*k*=6 instruments, n=8 studies) [11,13,20,21,27,28,40,42], allied health professions (*k*=5 instruments, n=5 studies) [9,22,35,36,38], midwifery (*k*=2 instruments, n=3 studies) [10,32,33], and dentistry (*k*=1 instrument, n=1 study) [37]. One instrument was explicitly described as a holistic, interprofessional tool for use across multiple healthcare disciplines [9].

Geographically, research was primarily concentrated in Australia (n=16) [8,10,14,15,22-26,29,32,33,36,38,39,41] and Europe, including Finland, Sweden, the United Kingdom, Germany, and the Netherlands (n=12) [12,17-20,27,28,30,31,35,37,42]. Additional studies were conducted in North America, including the United States and Canada (n=4) [16,40,45,46]; Asia, including Saudi Arabia, Thailand, Japan, China, and South Korea (n=6) [11,13,21,34,43,44]; and Africa, represented by South Africa (n=1) [9].

Most studies used cross-sectional psychometric validation designs or multi-stage instrument development phases that included literature reviews, qualitative interviews, or expert Delphi panels to establish content validity. Detailed characteristics of all included instruments and studies are provided in Dataset 1.

### Risk of bias in studies

For 9 tools, we did not conduct a new risk of bias assessment: 6 had been rated in a prior review [4], 1 was unavailable in full text [27], and 2 were non-English [43,44].

In this review, no standalone content validity studies were identified; evidence for content validity was derived from original instrument development studies.

Overall, most studies were rated as inadequate (*k*=21), primarily because of flaws in concept elicitation (*k*=8), missing pilot testing, and/or a lack of target population involvement (*k*=13). Specific findings for the development process are provided below.

Concept elicitation (Box 1a): Eight tools were rated as inadequate (Interprofessional Clinical Placement Learning Environment Inventory [ICPLEI], Quality Clinical Placement Evaluation [QCPE], Manchester Clinical Placement Index [MCPI], Ascent to Competence Scale [ACS], Clinical Placement Quality Survey–Student [CPQS-S], Clinical Learning Climate Measure [CLCM], holistic clinical learning environment measuring instrument [HM], and Quality Practical Experience [QPE]), and 9 were rated as doubtful (Clinical Learning Evaluation Questionnaire [CLEQ], Placement Evaluation Tool [PET], Dental Clinical Learning Environment Instrument [DECLEI], Placement Quality Measure [PQM], Health Education Learning Environment Survey [HELES], Undergraduate Clinical Education Environment Measure [UCEEM], Healthcare Education Micro Learning Environment Measure [HEMLEM], Clinical Placement Evaluation Tool [CPET], and Placement Evaluation Tool for Midwifery [PET-Midwifery]). Only one tool achieved an adequate rating (Midwifery Student Evaluation of Practice [MidSTEP]), and none was rated as very good.

Pilot testing (Box 1b): Thirteen tools had no pilot testing or were rated as inadequate (CLEQ, ICPLEI, QCPE, MCPI, PQM, ACS, HELES, HEMLEM, CPQS-S, CPET, CLCM, HM, and QPE). Five tools were rated as doubtful (PET-Midwifery, UCEEM, DECLEI, MidSTEP, and PET), while none reached the threshold for an adequate or very good risk of bias rating.

Overall, only 5 tools were rated as doubtful for Box 1 (DECLEI, PET, PET-Midwifery, MidSTEP, and UCEEM), and none were rated as adequate or very good.

A comprehensive breakdown of the risk of bias for each study is available in the supplementary material (Dataset 2. COSMIN file with risk of bias and content validity ratings).

### Results of syntheses

#### Content validity

Applying a content-validity-first approach, only 4 instruments (PET, PET-Midwifery, DECLEI, and MidSTEP) demonstrated sufficient evidence across relevance, comprehensiveness, and comprehensibility. All remaining 24 instruments were rated as insufficient, primarily because of indeterminate evidence for comprehensibility or inadequate reporting of the development process. Consequently, additional measurement properties were assessed only for these 4 instruments and are listed in Table 1 [10,25,32,33,37].

#### Clinical learning environment tools

Placement Evaluation Tool: The PET was developed by Cooper et al. [25] in 2020 specifically for nursing students in Australia. It consists of 19 items scored on a 5-point Likert scale, plus one global satisfaction rating. The items are organized into 2 factors: clinical environment and learning support. Although the tool demonstrated high internal consistency (Cronbach’s alpha values of 0.94 and 0.96) and sufficient content validity based on moderate-quality evidence, its structural validity was rated as insufficient. This rating was due to factor loading patterns in the exploratory factor analysis (EFA) that showed substantial cross-loadings, indicating that the dimensionality was not adequately supported. Consequently, its internal consistency and reliability were rated as indeterminate, as these properties cannot be meaningfully interpreted without a supported factor structure.

Placement Evaluation Tool–Midwifery: The PET-Midwifery is an adaptation of the original PET, with language adapted to the midwifery profession and its specific clinical contexts [10]. Developed by Bogossian et al. [10] in 2025, the tool maintains the 19-item structure and was evaluated with a national sample of 248 midwifery students across 7 Australian universities. The validation study established sufficient content validity with moderate-quality evidence, supported by a content validity index exceeding 0.90 for both relevance and clarity. However, like its parent version, it was rated as having insufficient structural validity and indeterminate internal consistency. Despite these psychometric ratings, the tool demonstrated strong concurrent validity with the MidSTEP scale.

Midwifery Student Evaluation of Practice: The MidSTEP tool is a multidimensional instrument designed for midwifery students, consisting of 26 items divided into 2 scales: the Clinical Learning Environment Scale (16 items) and the Impact of the Midwifery Preceptor Scale (10 items), each comprising 2 subscales [32]. Initially developed by Griffiths et al. [32] in 2020, it received moderate-quality evidence for sufficient content validity. MidSTEP’s structural validity and internal consistency are supported by a secondary study using Rasch analysis [33]. The Rasch analysis confirmed unidimensionality and established a sufficient internal consistency rating, with a PSI between 0.82 and 0.86.

Dental Clinical Learning Environment Instrument: The DECLEI was developed by Kossioni et al. [37] in 2014 to assess the CLE of undergraduate dental students. It consists of 24 items organized into 3 factors: organization and learning opportunities, professionalism and communication, and satisfaction and personal commitment. The tool achieved a sufficient rating for content validity with moderate-quality evidence. However, structural validity was rated as insufficient because the resulting solution did not meet COSMIN’s strict criteria for dimensionality, as only 43% of the variance in the tool could be explained by the 3 factors. Because dimensionality was not adequately established, the tool’s reported high internal consistency (α=0.89) was rated as indeterminate. The quality of this evidence was judged to be very low because the factor analytic study used principal component analysis (PCA), yielding a doubtful risk of bias rating, and because the sample size was too small to support the statistical power of the method used, yielding an inadequate risk of bias rating [5].

#### Re-evaluation of previously reported instruments

One instrument, SECEE version 4, previously rated as having sufficient content validity in earlier reviews, was re-evaluated in the present study.

Student Evaluation of the Clinical Education Environment (SECEE): The SECEE version 4 is a 35-item inventory originally developed by Sand-Jecklin [45] in 2009 and further refined in subsequent studies for undergraduate nursing students in the United States [46]. It includes 3 subscales: instructor facilitation of learning, preceptor facilitation of learning, and learning opportunities. Although a previous systematic review recommended the SECEE with a Grade A rating, the current review conducted a re-evaluation that led to a different conclusion [4]. Comprehensibility was not established through direct assessment with the target population during development. Consequently, the instrument’s content validity was rated as insufficient at the tool level, and the tool was excluded from the final recommendation summary table because of this foundational lack of primary evidence for comprehensibility.

### Reporting biases

For all included studies, the measurement properties specified in the methods sections were systematically compared with those reported in the results. Across the included studies, internal consistency and structural validity were reported whenever these properties were specified a priori. No instances were identified in which prespecified measurement properties were omitted from the results sections.

In addition, no patterns were observed in which favorable psychometric results were selectively reported while nonsignificant or unfavorable findings were omitted. However, none of the included studies reported preregistered study protocols, and exploratory psychometric analyses predominated. Consequently, assessment of reporting bias was limited to within-publication consistency between stated methods and reported results.

### Certainty of evidence

High-quality evidence supported sufficient structural validity and internal consistency for MidSTEP based on Rasch analysis [33]. In contrast, structural validity for PET, PET-Midwifery, and DECLEI was rated as insufficient, with moderate to very low quality of evidence, primarily because of methodological limitations in factor analytic approaches [10,25], sample size [37], and concerns regarding dimensionality [10,25].

Internal consistency was rated as indeterminate for PET, PET-Midwifery, and DECLEI because structural validity was insufficient; therefore, no certainty of evidence was assigned for this property in accordance with COSMIN guidelines.

## Discussion

### Interpretation/comparison with previous studies

This review identified 28 distinct CLE instruments across health professions, yet only 4 demonstrated sufficient content validity: the PET, the PET-Midwifery, the MidSTEP, and the DECLEI. This finding indicates that CLE instruments with sufficient evidence for measurement properties remain scarce and that substantial opportunities exist to advance the measurement and improvement of CLE quality.

Although the initial database search yielded 8,551 records and 39 studies were ultimately included, the number of tools meeting COSMIN content validity criteria was very limited. The excellent inter-rater agreement achieved in both title and abstract screening (κ=0.98) and full-text screening (κ=0.89) supports the reliability of the selection process. Despite the breadth of available instruments across nursing, medicine, allied health, dentistry, and midwifery, most tools did not demonstrate adequate evidence of content validity. This reinforces the need for more rigorous instrument development and more systematic approaches to content validation in future CLE research.

A major reason for insufficient content validity ratings was the lack of involvement of the target population during concept elicitation or the absence of comprehensibility testing during item development. According to COSMIN, comprehensibility testing is essential to ensure that items are interpreted as intended [5]. Without clear evidence of comprehensibility, students may understand items differently than anticipated, which can distort scale scores and undermine the validity of findings derived from these instruments.

Among the 4 tools with sufficient content validity, only the MidSTEP instrument allowed structural validity and internal consistency to be meaningfully interpreted. Its unidimensionality was supported by Rasch analysis, which generated high-quality evidence for structural validity and internal consistency. In contrast, the PET, PET-Midwifery, and DECLEI instruments lacked sufficient structural validity, which prevented meaningful interpretation of internal consistency coefficients. COSMIN guidelines specify that internal consistency can be interpreted only when unidimensionality is established [5]. Even when items are conceptually relevant and clear, insufficient structural validity creates uncertainty regarding whether a score reflects a coherent underlying construct. Moreover, a lack of structural validity limits understanding of how the items function psychometrically as a scale. Consequently, the content of the instrument can be trusted in terms of content validity, but it remains uncertain whether the score is statistically stable and whether the tool is reliable.

The review also identified significant methodological issues in the included studies. Several authors used PCA while referring to it as EFA. PCA is primarily a data reduction method and does not model latent constructs in the same way as common factor analysis [47]. This misapplication often resulted in inappropriate dimensionality conclusions. The PET instrument illustrates this issue. Although a 2-component PCA solution was extracted, substantial cross-loadings were found, suggesting the potential presence of a general factor rather than separable dimensions. A more appropriate re-analysis using latent variable modeling could clarify the true structure of the instrument.

Terminological inconsistencies also emerged. Cronbach’s alpha was frequently labeled as “reliability,” even though COSMIN defines reliability as the proportion of true variance estimated through test-retest or inter-rater designs. Cronbach’s alpha measures only internal consistency [5]. Misinterpretations of this kind can lead to inaccurate conclusions about an instrument’s psychometric robustness.

The widespread use of certain instruments does not necessarily reflect psychometric rigor. The CLES+T scale, for example, is widely used internationally [4], yet recent evaluations have identified inconsistent evidence for its content validity. This underscores the need to avoid equating popularity with psychometric adequacy.

### Relationship to discourses on quality in higher education and health professions education

From a discursive perspective, notions of quality in higher and health professions education are shaped by different discursive logics. Walter et al. [2] in 2026 describe 3 dominant perspectives: quality assurance (QA), emphasizing standardization and accountability; total quality management (TQM), focusing on efficiency and performance; and quality culture (QC), highlighting shared values, participation, and organizational learning. These perspectives influence which aspects of quality become visible, are operationalized, and are ultimately measured.

The findings of this review reflect this landscape. Most CLE instruments prioritize supervisory support, interpersonal relationships, and structural features of placements—dimensions closely aligned with QA-oriented approaches. Although relational aspects partly reflect QC perspectives, broader elements of organizational learning are largely absent. TQM-related dimensions, such as efficiency or strategic improvement, are not explicitly operationalized.

Overall, CLE measurement appears anchored in QA-oriented and interpersonal conceptualizations, with limited representation of cultural and organizational dimensions. These gaps likely reflect not only methodological limitations but also discursive assumptions about what constitutes quality in the CLE. Future instrument development should therefore more explicitly incorporate perspectives on organizational learning and participatory culture, which are closely linked to competence development.

### Comparison with previous studies

Compared with previous reviews in nursing and allied health professions, the present review applied a substantially more rigorous lens by adhering to COSMIN standards. Earlier reviews catalogued instruments but did not systematically evaluate content validity, structural validity, or reliability [3,4]. Consequently, their recommendations often relied on descriptive statistics or insufficiently evaluated psychometric claims. This review demonstrates that instruments previously recommended in the literature did not meet COSMIN criteria for CLE-specific content validity. The re-evaluation of SECEE version 4 illustrates this point. Although previously rated as a Grade A instrument, it lacked empirical assessment of comprehensibility with the target population and therefore did not meet COSMIN criteria for sufficient content validity at the tool level. This underscores the importance of conceptual clarity and methodological rigor when evaluating CLE instruments.

### Limitations

Despite its methodological rigor, this review has limitations. Although EMBASE and the Education Database were initially included as information sources in the review protocol, both were excluded from the final search. In the case of EMBASE, adaptation of the MEDLINE-based strategy using EMTREE terms resulted in a disproportionate number of records, with over 36,000 compared with 4,600 in MEDLINE, largely because of EMBASE’s broader indexing of pharmacological and unrelated clinical studies outside the scope of this review. Including EMBASE would have significantly reduced search specificity and increased screening burden without evident benefit for identifying additional relevant tools. The Education Database was excluded because of a lack of access.

This limitation was mitigated by comprehensive supplementary searches and by the use of multiple overlapping databases. The use of the SPIDER framework may have introduced bias by narrowing the scope of retrieved studies, although iterative refinement helped broaden the search. Most CLE instruments were developed using a psychometric-first rather than content-validity-first logic, which COSMIN considers suboptimal. Reviewer-based assessments of relevance, comprehensiveness, and comprehensibility were intentionally not applied, as they cannot replace empirical involvement of the target population.

Another limitation of this review is its restriction to English-language publications, which may have introduced language bias and led to the exclusion of relevant studies published in other languages.

### Implications

The findings of this systematic review have important implications for CE research, practice, and governance in undergraduate health professions education. Although only 4 of the 28 identified instruments demonstrated sufficient content validity, and only one of these (MidSTEP) also showed sufficient structural validity, these instruments can be used in practice when their psychometric strengths and limitations are considered.

For practical application, instruments with sufficient content validity but insufficient structural validity may be appropriate for formative purposes, such as identifying areas for improvement, conducting needs assessments, or supporting quality enhancement initiatives. In contrast, MidSTEP currently provides the most robust basis for evaluative or comparative applications due to its established dimensionality and internal consistency. Educators should therefore carefully align instrument selection with the intended purpose of assessment.

### Suggestions for further studies

For future development, these findings emphasize the need for instrument developers to adhere closely to COSMIN guidelines and, in particular, to ensure direct involvement of the target population during concept elicitation and comprehensibility assessment. For instruments with limited or inconsistent evidence, renewed content validity studies and more rigorous psychometric analyses are warranted, especially regarding structural validity and reliability. The limited number of instruments with sufficient content validity also highlights the need for new or adapted tools in underrepresented disciplines such as physiotherapy.

Furthermore, the 4 instruments with sufficient content validity provide a foundation for cross-professional adaptation studies. Such work could determine whether the constructs and item formulations are applicable across different health professions and whether cultural or contextual adjustments are necessary. Given that internships constitute a substantial portion of the curriculum in many health professions programs, the use of evaluated instruments with sufficient evidence for measurement properties is essential to ensure high-quality learning environments and support students’ competence development.

## Conclusion

This systematic review identified 28 instruments designed to measure the CLE in undergraduate health professions education. Of these, 4 instruments (PET, PET-Midwifery, MidSTEP, and DECLEI) demonstrated sufficient content validity, and one instrument, MidSTEP, additionally showed sufficient structural validity. These findings indicate that although only a few CLE instruments meet COSMIN standards, the available tools can be used in practice. However, their use requires careful consideration of their psychometric strengths and limitations. Instruments with sufficient content validity but insufficient structural validity may be appropriate for formative evaluations, descriptive purposes, or quality improvement initiatives, whereas MidSTEP currently offers the most robust basis for evaluative or comparative applications.

Taken together, the results emphasize both what is currently available and what is still missing. They provide evidence-based guidance on which tools can be used confidently, which tools require further refinement, and where additional research is needed to establish sound content and structural validity. The review also highlights the potential for cross-professional adaptation and the need for new or improved instruments in underrepresented disciplines. Strengthening the methodological and conceptual foundations of CLE measurement remains essential for ensuring high-quality CE and supporting students’ competence development across health professions.

## Figures and Tables

**Figure f2-jeehp-23-14:**
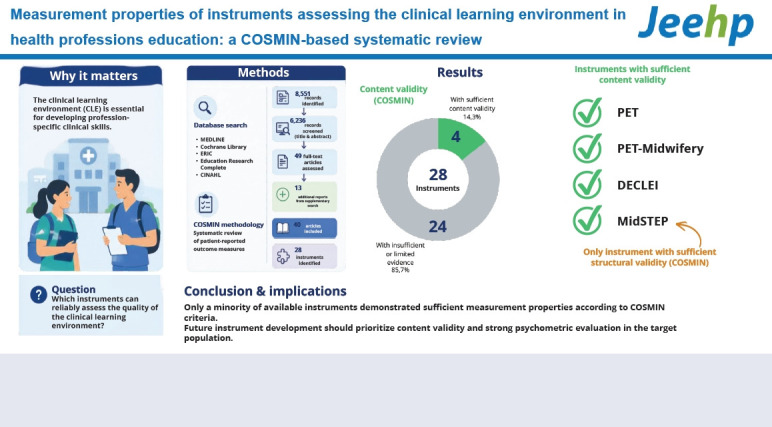


**Table 1.** Overview of tools with sufficient content validity

Instrument	Population	Author	Year	Items/factors	Content validity	Structural validity	Internal consistency (PSI)	Interpretation
PET	Nursing students (Australia)	Cooper et al. [25]	2020	19 items/2 factors	+ (Moderate)	− (Moderate)	?	Internal consistency uninterpretable due to insufficient structural validity
MidSTEP	Undergraduate midwifery students (Australia & NZ)	Griffiths et al. [32]; Gamble et al. [33]	2020/2022	26 items/2 factors with 2 subscales each	+ (Moderate)	+ (High)	+ (High)	Structural validity supported by Rasch analysis; internal consistency interpretable and sufficient
							Skill development: 0.82	
							Philosophy: 0.85	
							Preceptor skill: 0.84	
							Preceptor philosophy: 0.86	
DECLEI	Clinical dental students (multinational sample)	Kossioni et al. [37]	2014	24 items/3 factors	+ (Moderate)	− (Very low)	?	Internal consistency uninterpretable due to insufficient structural validity
PET-Midwifery	Midwifery students	Bogossian et al. [10]	2025	19 items/2 factors	+ (Moderate)	− (Moderate)	?	Internal consistency uninterpretable due to insufficient structural validity

PSI values are reported based on Rasch analysis. Certainty of evidence was rated using the COSMIN-adapted GRADE approach and is shown in brackets (high/moderate/low/very low). Internal consistency is interpreted only when structural validity is sufficient, in accordance with COSMIN recommendations. If structural validity is insufficient or indeterminate, internal consistency is rated as indeterminate and no certainty grading is applied. Rasch-based evidence (MidSTEP 2022) was prioritized over earlier factor analytic evidence following COSMIN guidance to synthesize the best available methodological evidence. PSI, person separation index; PET, Placement Evaluation Tool; +, sufficient; −, insufficient; ?, indeterminate according to the COSMIN criteria for good measurement properties; MidSTEP, Midwifery Student Evaluation of Practice; DECLEI, Dental Clinical Learning Environment Instrument; PET-Midwifery, Placement Evaluation Tool for Midwifery; COSMIN, Consensus-Based Standards for the Selection of Health Measurement Instruments; GRADE, Grading of Recommendations Assessment, Development and Evaluation.

## Footnotes

**Data availability**
Data files are available from https://doi.org/10.7910/DVN/KVISZQ Dataset 1. Characteristics of tools used in the literature.

Dataset 2. Consensus-Based Standards for the Selection of Health Measurement Instruments data file with risk of bias and content validity ratings.

## Supplementary materials

Supplement 1. Search strategy.

Supplement 2. Audio recording of the abstract.
