# Supplementary material for: Measurement properties of instruments assessing the clinical learning environment in health professions education: a COSMIN-based systematic review
Source: J Educ Eval Health Prof. 2026 Jun 11;23:14. doi: 10.3352/jeehp.2026.23.14 (PMC13472927; doi:10.3352/jeehp.2026.23.14)
Supplement: Supplementary file 1 [file jeehp-23-14-suppl1.docx]

Ovid MEDLINE(R) ALL <1946 to May 16, 2025>

1	"physical therapy modalities".sh.	42782

2	exp "Physical Therapists"/	4019

3	"Nurses".sh.	49038

4	exp "Allied Health Occupations"/	55330

5	exp "Nursing"/	269837

6	exp "Dietetics"/	8462

7	"Pharmacology".sh.	33597

8	exp "Students, Health Occupations"/	97661

9	exp "Chiropractic"/	3566

10	exp "Students, Nursing"/	33876

11	exp "Occupational Therapy"/	15940

12	exp "Occupational Therapists"/	951

13	exp "Speech Therapy"/	7212

14	exp "Nutritionists"/	2068

15	exp "Pharmacists"/	23909

16	exp "Students, Pharmacy"/	5116

17	exp "Students, Dental"/	8015

18	"Dentistry".sh.	36750

19	"Respiratory Therapy".sh.	7157

20	exp "Medical Laboratory Personnel"/	2801

21	exp "Students, Medical"/	48684

22	"Health Personnel".sh.	73577

23	"physical therapy modalities".ti,ab,kw.	1143

24	"physical therap*".ti,ab,kw.	38057

25	"physiotherap*".ti,ab,kw.	40494

26	"Nurs*".ti,ab,kw.	579491

27	"Allied Health Occupation*".ti,ab,kw.	98

28	"occupational therap*".ti,ab,kw.	20964

29	"health occupation student*".ti,ab,kw.	10

30	"Chiropract*".ti,ab,kw.	7335

31	"Dietetics".ti,ab,kw.	4019

32	"dietitian*".ti,ab,kw.	9171

33	"dietician*".ti,ab,kw.	2566

34	"Pharmacolog*".ti,ab,kw.	484839

35	"pharmacist*".ti,ab,kw.	48860

36	"nurs* student*".ti,ab,kw.	25463

37	"speech therap*".ti,ab,kw.	4935

38	"respiratory therap*".ti,ab,kw.	3588

39	"medical technolog*".ti,ab,kw.	7931

40	"health profession student*".ti,ab,kw.	308

41	"healthcare student*".ti,ab,kw.	1466

42	"pharmacy student*".ti,ab,kw.	4434

43	"dental student*".ti,ab,kw.	8979

44	"dentist*".ti,ab,kw.	96422

45	"nutrition*".ti,ab,kw.	416775

46	"Medical Laboratory Personnel".ti,ab,kw.	38

47	"medical student*".ti,ab,kw.	61304

48	"health occupation*".ti,ab,kw.	854

49	"health personnel".ti,ab,kw.	9620

50	exp "Education, Medical, Undergraduate"/	29253

51	"Education, Medical, Undergraduate".ti,ab,kw.	81

52	exp "Nursing Education Research"/	10283

53	"Nursing Education Research".ti,ab,kw.	413

54	exp "Education, Nursing, Baccalaureate"/	23318

55	"Education, Nursing, Baccalaureate".ti,ab,kw.	12

56	exp "Education, Nursing, Diploma Programs"/	3551

57	"Education, Nursing, Diploma Programs".ti,ab,kw.	0

58	"Education, Dental".sh.	16560

59	"Education, Pharmacy".ti,ab,kw.	200

60	"Education, Pharmacy".sh.	8291

61	"Pharmacy Residencies".sh.	454

62	"Pharmacy Residencies".ti,ab,kw.	100

63	"Education, Dental".ti,ab,kw.	624

64	"health student".ti,ab,kw.	183

65	"physical therap* student*".ti,ab,kw.	652

66	"physiotherap* student*".ti,ab,kw.	800

67	"respiratory care".ti,ab,kw.	2574

68	"respiratory therapy student*".ti,ab,kw.	52

69	"clinical laboratory personnel".ti,ab,kw.	59

70	"health care personnel".ti,ab,kw.	3306

71	"medical education".ti,ab,kw.	65877

72	"medical education".ti,ab,kw.	65877

73	"nursing education".ti,ab,kw.	19699

74	"dental student*".ti,ab,kw.	8979

75	"undergraduate student*s".ti,ab,kw.	13556

76	"Education, Nursing, Research-Based".ti,ab,kw.	0

77	"Education, Medical".ti,ab,kw.	6576

78	"Laboratory Personnel".ti,ab,kw.	1645

79	"ALLIED health personnel".ti,ab,kw.	389

80	1 or 2 or 3 or 4 or 5 or 6 or 7 or 8 or 9 or 10 or 11 or 12 or 13 or 14 or 15 or 16 or 17 or 18 or 19 or 20 or 21 or 22 or 23 or 24 or 25 or 26 or 27 or 28 or 29 or 30 or 31 or 32 or 33 or 34 or 35 or 36 or 37 or 38 or 39 or 40 or 41 or 42 or 43 or 44 or 45 or 46 or 47 or 48 or 49 or 50 or 51 or 52 or 53 or 54 or 55 or 56 or 57 or 58 or 59 or 60 or 61 or 62 or 63 or 64 or 65 or 67 or 68 or 69 or 70 or 71 or 72 or 73 or 74 or 75 or 76 or 77 or 78 or 79	2172760

81	exp "Clinical Clerkship"/	6041

82	"Clinical Clerkship".ti,ab,kw.	795

83	exp "Mentoring"/	4999

84	"Mentoring".ti,ab,kw.	10797

85	exp "Preceptorship"/	5876

86	"Preceptorship".ti,ab,kw.	1355

87	exp "Internship, Nonmedical"/	2222

88	"Internship, Nonmedical".ti,ab,kw.	7

89	exp "Internship and Residency"/	65327

90	"Internship and Residency".ti,ab,kw.	1273

91	"Teaching rounds".sh.	1617

92	"Teaching Round*".ti,ab,kw.	206

93	"Internship*".ti,ab,kw.	6574

94	"Clinical placement*".ti,ab,kw.	3079

95	"Clinical education".ti,ab,kw.	4152

96	"Clinical training*".ti,ab,kw.	6250

97	"Practicum".ti,ab,kw.	1584

98	"Clinical rotation*".ti,ab,kw.	2133

99	"Clinical experience*".ti,ab,kw.	52705

100	"Professional practice".ti,ab,kw.	7304

101	"Work-based learning".ti,ab,kw.	272

102	"Workplace learning".ti,ab,kw.	574

103	"Clinical supervision".ti,ab,kw.	2107

104	"Clinical teaching".ti,ab,kw.	3216

105	"Fieldwork".ti,ab,kw.	5268

106	"Experiential learning".ti,ab,kw.	3675

107	"Hands-on training".ti,ab,kw.	1529

108	"Apprenticeship*".ti,ab,kw.	1734

109	"educational setting*".ti,ab,kw.	2773

110	"residency education".ti,ab,kw.	1169

111	"mentorship".ti,ab,kw.	8756

112	"Interns and Residents".ti,ab,kw.	554

113	"educational setting*".ti,ab,kw.	2773

114	"Clinical learning environment*".ti,ab,kw.	1135

115	"learning environment*".ti,ab,kw.	9943

116	"Professional Autonomy".sh,ti,ab,kw.	10488

117	"learning setting*".ti,ab,kw.	552

118	"quality".ti,ab,kw.	1605317

119	113 or 114 or 115 or 116 or 117 or 118	1625908

120	81 or 82 or 83 or 84 or 85 or 86 or 87 or 88 or 89 or 90 or 91 or 92 or 93 or 94 or 95 or 96 or 97 or 98 or 99 or 100 or 101 or 102 or 103 or 104 or 105 or 106 or 107 or 108 or 109 or 110 or 111 or 112	188792

121	119 and 120	25120

122	"Focus Group*".sh,ti,ab,kw.	81943

123	"Reliability".ti,ab,kw.	250596

124	"Observation*".ti,ab,kw.	1150615

125	"Cohort".ti,ab,kw.	954057

126	"Surve*".ti,ab,kw.	1213447

127	"Interview*".ti,ab,kw.	519625

128	"Validation".ti,ab,kw.	377377

129	122 or 123 or 124 or 125 or 126 or 127 or 128	3998576

130	"Instrument*".ti,ab,kw.	385114

131	"Questionnaire*".ti,ab,kw.	767406

132	"Tool*".ti,ab,kw.	1166043

133	"Survey*".ti,ab,kw.	963816

134	"Scale*".ti,ab,kw.	1292495

135	"Inventor*".ti,ab,kw.	122804

136	"Evaluat*".ti,ab,kw.	5012697

137	"Assess*".ti,ab,kw.	4413731

138	"Measur*".ti,ab,kw.	4564905

139	"percept*".ti,ab,kw.	439654

140	"perspective*".ti,ab,kw.	543057

141	"view*".ti,ab,kw.	604752

142	130 or 131 or 132 or 133 or 134 or 135 or 136 or 137 or 138 or 139 or 140 or 141	13148999

143	80 and 121 and 129 and 142	4826

144	limit 143 to (english language and "remove preprint records")	**4702 results**

ERIC <1965 to April 2025>

1	"Physical Therapy".mp.	1425

2	"Nurses".mp.	7542

3	"Allied Health Occupations".mp.	6761

4	"Nursing".mp.	12437

5	"Dietetics".mp.	2220

6	"Pharmacology".mp.	1151

7	"Occupational Therapy".mp.	1914

8	"Speech Therapy".mp.	2538

9	"Dentistry".mp.	1644

10	"Respiratory Therapy".mp.	99

11	"Health Personnel".mp.	7648

12	"physical therapy modalities".ti,ab.	1

13	"physical therap*".ti,ab.	1091

14	"physiotherap*".ti,ab.	332

15	"Nurs*".ti,ab.	18074

16	"Allied Health Occupation*".ti,ab.	93

17	"occupational therap*".ti,ab.	1771

18	"health occupation student*".ti,ab.	3

19	"Chiropract*".ti,ab.	98

20	"Dietetics".ti,ab.	177

21	"dietitian*".ti,ab.	139

22	"dietician*".ti,ab.	59

23	"Pharmacolog*".ti,ab.	1027

24	"pharmacist*".ti,ab.	537

25	"nurs* student*".ti,ab.	2264

26	"speech therap*".ti,ab.	614

27	"respiratory therap*".ti,ab.	134

28	"medical technolog*".ti,ab.	333

29	"health profession student*".ti,ab.	24

30	"healthcare student*".ti,ab.	99

31	"pharmacy student*".ti,ab.	439

32	"dental student*".ti,ab.	388

33	"dentist*".ti,ab.	1378

34	"nutrition*".ti,ab.	9609

35	"Medical Laboratory Personnel".ti,ab.	10

36	"medical student*".ti,ab.	4140

37	"health occupation*".ti,ab.	1213

38	"health personnel".ti,ab.	415

39	"Education, Medical, Undergraduate".ti,ab.	0

40	"Nursing Education Research".ti,ab.	15

41	"Education, Nursing, Baccalaureate".ti,ab.	0

42	"Education, Nursing, Diploma Programs".ti,ab.	0

43	"Dental students (2004)".mp.	0

44	"Education, Pharmacy".ti,ab.	3

45	"Pharmacy".mp.	2195

46	"Pharmacy Residencies".sh.	0

47	"Pharmacy Residencies".ti,ab.	0

48	"Education, Dental".ti,ab.	20

49	"health student".ti,ab.	64

50	"nursing student".ti,ab.	270

51	"respiratory care".ti,ab.	58

52	"respiratory therapy student*".ti,ab.	2

53	"clinical laboratory personnel".ti,ab.	8

54	"health care personnel".ti,ab.	83

55	"medical education".ti,ab.	3297

56	"medical education".ti,ab.	3297

57	"nursing education".ti,ab.	1654

58	"undergraduate students".mp.	60086

59	"allied health personnel".ti,ab.	50

60	"Laboratory Personnel".ti,ab.	48

61	"Education, Medical".ti,ab.	126

62	"Education, Nursing, Research-Based".ti,ab.	0

63	1 or 2 or 3 or 4 or 5 or 6 or 7 or 8 or 9 or 10 or 11 or 12 or 13 or 14 or 15 or 16 or 17 or 18 or 19 or 20 or 21 or 22 or 23 or 24 or 25 or 26 or 27 or 28 or 29 or 30 or 31 or 32 or 33 or 34 or 35 or 36 or 37 or 38 or 39 or 40 or 41 or 42 or 43 or 44 or 45 or 46 or 47 or 48 or 49 or 50 or 51 or 52 or 53 or 54 or 55 or 56 or 57 or 58 or 59 or 60 or 61 or 62	114294

64	"Clinical Experience".sh.	3630

65	"Clinical Clerkship".ti,ab.	60

66	"Mentoring".ti,ab.	13019

67	"Preceptorships".sh.	0

68	"Preceptorship".ti,ab.	79

69	"Internship Programs".sh.	5608

70	"Internship, Nonmedical".ti,ab.	0

71	"Graduate Medical Education".sh.	1372

72	"Internship and Residency".ti,ab.	35

73	"Teaching rounds".sh.	0

74	"Teaching Round*".ti,ab.	26

75	"Internship*".ti,ab.	7292

76	"Clinical placement*".ti,ab.	246

77	"Clinical education".ti,ab.	452

78	"Clinical training*".ti,ab.	450

79	"Practicum".ti,ab.	5954

80	"Clinical rotation*".ti,ab.	113

81	"Clinical experience*".ti,ab.	1583

82	"Professional practice".ti,ab.	3483

83	"Work-based learning".ti,ab.	1298

84	"Workplace learning".ti,ab.	1223

85	"Clinical supervision".ti,ab.	695

86	"Clinical teaching".ti,ab.	295

87	"Fieldwork".ti,ab.	2970

88	"Experiential learning".ti,ab.	5967

89	"Hands-on training".ti,ab.	263

90	"Apprenticeship*".ti,ab.	4586

91	"educational setting*".ti,ab.	7088

92	"residency education".ti,ab.	28

93	"mentorship".ti,ab.	2894

94	"Interns and Residents".ti,ab.	25

95	"educational setting*".ti,ab.	7088

96	"Clinical learning environment*".ti,ab.	43

97	"learning environment*".mp.	32545

98	"Professional Autonomy".mp.	3894

99	"learning setting*".ti,ab.	1373

100	"quality".ti,ab.	117236

101	95 or 96 or 97 or 98 or 99 or 100	157355

102	64 or 65 or 66 or 67 or 68 or 69 or 70 or 71 or 72 or 73 or 74 or 75 or 76 or 77 or 78 or 79 or 80 or 81 or 82 or 83 or 84 or 85 or 86 or 87 or 88 or 89 or 90 or 91 or 92 or 93 or 94	59737

103	101 and 102	13002

104	"Focus Group*".mp.	26634

105	"Reliability".ti,ab.	24974

106	"Observation*".ti,ab.	66785

107	"Cohort".ti,ab.	12640

108	"Surve*".ti,ab.	192260

109	"Interview*".ti,ab.	169691

110	"Validation".ti,ab.	13366

111	104 or 105 or 106 or 107 or 108 or 109 or 110	416215

112	"Instrument*".ti,ab.	69600

113	"Questionnaire*".ti,ab.	98861

114	"Tool*".ti,ab.	101665

115	"Survey*".ti,ab.	190693

116	"Scale*".ti,ab.	85834

117	"Inventor*".ti,ab.	24853

118	"Evaluat*".ti,ab.	255104

119	"Assess*".ti,ab.	258687

120	"Measur*".ti,ab.	194141

121	"percept*".ti,ab.	124369

122	"perspective*".ti,ab.	125923

123	"view*".ti,ab.	121241

124	112 or 113 or 114 or 115 or 116 or 117 or 118 or 119 or 120 or 121 or 122 or 123	998881

125	63 and 103 and 111 and 124	505

126	limit 125 to (english language and peer reviewed)	**325**

**CINAHL: Peer reviewed + english only: 2.340 results
S:**

(	(MH "Physical Therapy" OR TI "physical therapy modalities" OR AB "physical therapy modalities" OR TI "physical therap*" OR AB "physical therap*" OR TI "physiotherap*" OR AB "physiotherap*") OR	(MH "Physical Therapists" OR TI "physical therapist*" OR AB "physical therapist*") OR	(MH "Nurses" OR TI "nurs*" OR AB "nurs*" OR TI “NURSING education“ OR AB „NURSING education“) OR	(MH "Health Occupations" OR TI "allied health occupation*" OR AB "allied health occupation*" OR TI "health occupation*" OR AB "health occupation*") OR	(MH "Nursing" OR TI "nursing" OR AB "nursing") OR	(MH "Dietetics" OR TI "dietetics" OR AB "dietetics" OR TI "dietitian*" OR AB "dietitian*" OR TI "dietician*" OR AB "dietician*") OR	(MH "Pharmacology" OR TI "pharmacolog*" OR AB "pharmacolog*") OR	(MH "Students, Health Occupations" OR TI "health occupation student*" OR AB "health occupation student*" OR TI "health profession student*" OR AB "health profession student*" OR TI "healthcare student*" OR AB "healthcare student*") OR	(MH "Chiropractic" OR TI "chiropract*" OR AB "chiropract*") OR	(MH "Students, Nursing" OR TI "nurs* student*" OR AB "nurs* student*") OR	(MH "Occupational Therapy" OR TI "occupational therap*" OR AB "occupational therap*") OR	(MH "Occupational Therapists" OR TI "occupational therapist*" OR AB "occupational therapist*" OR TI "occupational therapist*" OR AB "occupational therapist*" OR TI “health occupation* student*“ OR AB “health occupation* student*“)) OR	(MH "Speech Therapy" OR TI "speech therap*" OR AB "speech therap*") OR	(MH "Nutritionists" OR TI "nutrition*" OR AB "nutrition*" OR TI "nutritionist*" OR AB "nutritionist*") OR	(MH "Pharmacists" OR TI "pharmacist*" OR AB "pharmacist*") OR	(MH "Students, Pharmacy" OR TI "pharmacy student*" OR AB "pharmacy student*") OR	(MH "Students, Dental" OR TI "dental student*" OR AB "dental student*") OR	(MH "Dentistry" OR TI "dentist*" OR AB "dentist*") OR	(MH "Respiratory Therapy" OR TI "respiratory therap*" OR AB "respiratory therap*" OR TI "respiratory care" OR AB "respiratory care" OR TI "respiratory therapy student*" OR AB "respiratory therapy student*") OR	(MH "Laboratory Personnel" OR TI "medical technolog*" OR AB "medical technolog*" OR TI "clinical laboratory personnel" OR AB "clinical laboratory personnel") OR	(MH "Students, Medical" OR TI "medical student*" OR AB "medical student*") OR	(MH "Health Personnel" OR TI "health personnel" OR AB "health personnel" OR TI "health care personnel" OR AB "health care personnel" OR TI “Allied health personnel“ OR AB “Allied health personnel“) OR	(MH "Education, Medical" OR TI "medical education" OR AB "medical education" OR TI "undergraduate student*" OR AB "undergraduate student*") OR	(MH "Education, Nursing, Research-Based" OR TI "nursing education research" OR AB "nursing education research") OR	(MH "Education, Nursing, Baccalaureate" OR TI "education, nursing, baccalaureate" OR AB "education, nursing, baccalaureate") OR	(MH "Education, Nursing, Diploma Programs" OR TI "education, nursing, diploma programs" OR AB "education, nursing, diploma programs") OR	(MH "Education, Dental" OR TI "education, dental" OR AB "education, dental") OR	(MH "Education, Pharmacy" OR TI "education, pharmacy" OR AB "education, pharmacy") OR	(MH "Pharmacy Residencies" OR TI "pharmacy residencies" OR AB "pharmacy residencies") OR	(TI "health student" OR AB "health student" OR TI "physical therap* student*" OR AB "physical therap* student*" OR TI "physiotherap* student*" OR AB "physiotherap* student*") )

**PI:**

((	(MH "Clinical Clerkship" OR TI "clinical clerkship" OR AB "clinical clerkship") OR	(MH "Mentorship" OR TI "mentoring" OR AB "mentoring") OR	(MH "Preceptorship" OR TI "preceptorship" OR AB "preceptorship") OR	(MH "Interns and Residents" OR TI "internship, nonmedical" OR AB "internship, nonmedical") OR	(MH "Internship and Residency" OR TI "internship and residency" OR AB "internship and residency") OR	(MH "Teaching Rounds" OR TI "teaching round*" OR AB "teaching round*") OR	(TI "internship*" OR AB "internship*") OR	(TI "clinical placement*" OR AB "clinical placement*") OR	(TI "clinical education" OR AB "clinical education") OR	(TI "clinical training*" OR AB "clinical training*") OR	(TI "practicum" OR AB "practicum") OR	(TI "clinical rotation*" OR AB "clinical rotation*") OR	(TI "clinical experience*" OR AB "clinical experience*") OR	(TI "professional practice" OR AB "professional practice") OR	(TI "work-based learning" OR AB "work-based learning") OR	(TI "workplace learning" OR AB "workplace learning") OR	(TI "clinical supervision" OR AB "clinical supervision") OR	(TI "clinical teaching" OR AB "clinical teaching") OR	(TI "fieldwork" OR AB "fieldwork") OR	(TI "experiential learning" OR AB "experiential learning") OR	(TI "hands-on training" OR AB "hands-on training") OR	(TI "apprenticeship*" OR AB "apprenticeship*") OR	(TI "educational setting*" OR AB "educational setting*") OR	(TI "residency education" OR AB "residency education") OR TI “INTERNSHIP programs“ OR AB “INTERNSHIP programs“ OR TI “NURSING preceptorship“ OR AB “NURSING preceptorship“ OR TI “ MEDICAL preceptorship“ OR AB “ MEDICAL preceptorship“

)) AND ((MH "Professional Autonomy" OR TI "Professional Autonomy" OR AB "Professional Autonomy") OR (TI "educational setting" OR AB "educational setting") OR (TI "Clinical learning environment" OR AB "Clinical learning environment") OR (TI "Clinical learning environment" OR AB "Clinical learning environment") OR (TI "learning environment*" OR AB "learning environment*") OR (TI "learning setting*" OR AB "learning setting*") OR ( TI “quality" OR AB "quality"))

**D:**

( TI "focus group*" OR AB "focus group*") OR ( TI "Reliability" OR AB "Reliability") OR ( TI "Observation*" OR AB "Observation*") OR ( TI "Cohort" OR AB "Cohort") OR ( TI "Surve*" OR AB "Surve*") OR ( TI "Interview*" OR AB "Interview*") OR ( TI "Validation" OR AB "Validation")

**E:**

( TI "Instrument*“ OR AB " Instrument*") OR ( TI "Questionnaire*" OR AB "Questionnaire*") OR ( TI "Tool*".OR AB "Tool*") OR ( TI "Surve*" OR AB "Surve*") OR ( TI "Scale*" OR AB "Scale*") OR ( TI "Inventor*" OR AB "Inventor*") OR ( TI "Evaluat*" OR AB "Evaluat*") OR ( TI "Assess*“ OR AB "Assess*") OR ( TI "Measur*" OR AB "Measur*") OR ( TI "percpt*".OR AB "percept*") OR ( TI "perspective*" OR AB "perspective*") OR ( TI "view*" OR AB "view*")

**ERC: 727 results (peer reviewed+ english)**

**S:**

( (MH "ALLIED health personnel" OR MH "HEALTH occupations students" OR MH "NURSING students" OR MH "PHARMACY students" OR MH "DENTAL students" OR MH "MEDICAL students" OR MH "NURSING education" OR MH "BACCALAUREATE nursing education" OR MH "DENTAL education" OR MH "PHARMACY education") OR (MH "Physical Therapy" OR TI "physical therapy modalities" OR AB "physical therapy modalities" OR TI "physical therap*" OR AB "physical therap*" OR TI "physiotherap*" OR AB "physiotherap*") OR	(MH "Physical Therapists" OR TI "physical therapist*" OR AB "physical therapist*") OR	(MH "Nurses" OR TI "nurs*" OR AB "nurs*" OR TI “NURSING education“ OR AB „NURSING education“) OR	(MH "Health Occupations" OR TI "allied health occupation*" OR AB "allied health occupation*" OR TI "health occupation*" OR AB "health occupation*") OR	(MH "Nursing" OR TI "nursing" OR AB "nursing") OR	(MH "Dietetics" OR TI "dietetics" OR AB "dietetics" OR TI "dietitian*" OR AB "dietitian*" OR TI "dietician*" OR AB "dietician*") OR	(MH "Pharmacology" OR TI "pharmacolog*" OR AB "pharmacolog*") OR	(MH "Students, Health Occupations" OR TI "health occupation student*" OR AB "health occupation student*" OR TI "health profession student*" OR AB "health profession student*" OR TI "healthcare student*" OR AB "healthcare student*") OR	(MH "Chiropractic" OR TI "chiropract*" OR AB "chiropract*") OR	(MH "Students, Nursing" OR TI "nurs* student*" OR AB "nurs* student*") OR	(MH "Occupational Therapy" OR TI "occupational therap*" OR AB "occupational therap*") OR	(MH "Occupational Therapists" OR TI "occupational therapist*" OR AB "occupational therapist*" OR TI "occupational therapist*" OR AB "occupational therapist*" OR TI “health occupation* student*“ OR AB “health occupation* student*“)) OR	(MH "Speech Therapy" OR TI "speech therap*" OR AB "speech therap*") OR	(MH "Nutritionists" OR TI "nutrition*" OR AB "nutrition*" OR TI "nutritionist*" OR AB "nutritionist*") OR	(MH "Pharmacists" OR TI "pharmacist*" OR AB "pharmacist*") OR	(MH "Students, Pharmacy" OR TI "pharmacy student*" OR AB "pharmacy student*") OR	(MH "Students, Dental" OR TI "dental student*" OR AB "dental student*") OR	(MH "Dentistry" OR TI "dentist*" OR AB "dentist*") OR	(MH "Respiratory Therapy" OR TI "respiratory therap*" OR AB "respiratory therap*" OR TI "respiratory care" OR AB "respiratory care" OR TI "respiratory therapy student*" OR AB "respiratory therapy student*") OR	(MH "Laboratory Personnel" OR TI "medical technolog*" OR AB "medical technolog*" OR TI "clinical laboratory personnel" OR AB "clinical laboratory personnel") OR	(MH "Students, Medical" OR TI "medical student*" OR AB "medical student*") OR	(MH "Health Personnel" OR TI "health personnel" OR AB "health personnel" OR TI "health care personnel" OR AB "health care personnel" OR TI “Allied health personnel“ OR AB “Allied health personnel“) OR	(MH "Education, Medical" OR TI "medical education" OR AB "medical education" OR TI "undergraduate student*" OR AB "undergraduate student*") OR	(MH "Education, Nursing, Research-Based" OR TI "nursing education research" OR AB "nursing education research") OR	(MH "Education, Nursing, Baccalaureate" OR TI "education, nursing, baccalaureate" OR AB "education, nursing, baccalaureate") OR	(MH "Education, Nursing, Diploma Programs" OR TI "education, nursing, diploma programs" OR AB "education, nursing, diploma programs") OR	(MH "Education, Dental" OR TI "education, dental" OR AB "education, dental") OR	(MH "Education, Pharmacy" OR TI "education, pharmacy" OR AB "education, pharmacy") OR	(MH "Pharmacy Residencies" OR TI "pharmacy residencies" OR AB "pharmacy residencies") OR	(TI "health student" OR AB "health student" OR TI "physical therap* student*" OR AB "physical therap* student*" OR TI "physiotherap* student*" OR AB "physiotherap* student*") )

**PI:**

((	(MH "Clinical Clerkship" OR TI "clinical clerkship" OR AB "clinical clerkship") OR	(MH "Mentorship" OR TI "mentoring" OR AB "mentoring") OR	(MH "Preceptorship" OR TI "preceptorship" OR AB "preceptorship") OR	(MH "Interns and Residents" OR TI "internship, nonmedical" OR AB "internship, nonmedical") OR	(MH "Internship and Residency" OR TI "internship and residency" OR AB "internship and residency") OR	(MH "Teaching Rounds" OR TI "teaching round*" OR AB "teaching round*") OR	(TI "internship*" OR AB "internship*") OR	(TI "clinical placement*" OR AB "clinical placement*") OR	(TI "clinical education" OR AB "clinical education") OR	(TI "clinical training*" OR AB "clinical training*") OR	(TI "practicum" OR AB "practicum") OR	(TI "clinical rotation*" OR AB "clinical rotation*") OR	(TI "clinical experience*" OR AB "clinical experience*") OR	(TI "professional practice" OR AB "professional practice") OR	(TI "work-based learning" OR AB "work-based learning") OR	(TI "workplace learning" OR AB "workplace learning") OR	(TI "clinical supervision" OR AB "clinical supervision") OR	(TI "clinical teaching" OR AB "clinical teaching") OR	(TI "fieldwork" OR AB "fieldwork") OR	(TI "experiential learning" OR AB "experiential learning") OR	(TI "hands-on training" OR AB "hands-on training") OR	(TI "apprenticeship*" OR AB "apprenticeship*") OR	(TI "educational setting*" OR AB "educational setting*") OR	(TI "residency education" OR AB "residency education") OR MH “INTERNSHIP programs“OR TI “INTERNSHIP programs“ OR AB “INTERNSHIP programs“ OR MH “NURSING preceptorship“OR TI “NURSING preceptorship“ OR AB “NURSING preceptorship“ OR MH “ MEDICAL preceptorship“OR TI “ MEDICAL preceptorship“ OR AB “ MEDICAL preceptorship“ )) AND ((MH "Professional Autonomy" OR TI "Professional Autonomy" OR AB "Professional Autonomy") OR (TI "educational setting" OR AB "educational setting") OR (TI "Clinical learning environment" OR AB "Clinical learning environment") OR (TI "Clinical learning environment" OR AB "Clinical learning environment") OR (TI "learning environment*" OR AB "learning environment*") OR (TI "learning setting*" OR AB "learning setting*") OR ( TI “quality" OR AB "quality"))

**D:**

( TI "focus group*" OR AB "focus group*") OR ( TI "Reliability" OR AB "Reliability") OR ( TI "Observation*" OR AB "Observation*") OR ( TI "Cohort" OR AB "Cohort") OR ( TI "Surve*" OR AB "Surve*") OR ( TI "Interview*" OR AB "Interview*") OR ( TI "Validation" OR AB "Validation")

**E:**

( TI "Instrument*“ OR AB " Instrument*") OR ( TI "Questionnaire*" OR AB "Questionnaire*") OR ( TI "Tool*".OR AB "Tool*") OR ( TI "Surve*" OR AB "Surve*") OR ( TI "Scale*" OR AB "Scale*") OR ( TI "Inventor*" OR AB "Inventor*") OR ( TI "Evaluat*" OR AB "Evaluat*") OR ( TI "Assess*“ OR AB "Assess*") OR ( TI "Measur*" OR AB "Measur*") OR ( TI "percpt*".OR AB "percept*") OR ( TI "perspective*" OR AB "perspective*") OR ( TI "view*" OR AB "view*")

**Cochrane: 495 results 🡪 453 reviews (extrahiert), 37 protocols (nicht extrahiert da ausgeschlossen),**

Search Name:	SR_VUB_25

Date Run:	19/05/2025 23:48:47

Comment:

ID	Search	Hits

#1	(

MH "Physical Therapy"

OR TI (physical NEXT therapy NEXT modalities)

OR AB (physical NEXT therapy NEXT modalities)

OR TI (physical NEXT therap*)

OR AB (physical NEXT therap*)

OR TI physiotherap*

OR AB physiotherap*

OR MH "Physical Therapists"

OR TI (physical NEXT therapist*)

OR AB (physical NEXT therapist*)

OR MH "Nurses"

OR TI nurs*

OR AB nurs*

OR TI (nursing NEXT education)

OR AB (nursing NEXT education)

OR MH "Health Occupations"

OR TI (allied NEXT health NEXT occupation*)

OR AB (allied NEXT health NEXT occupation*)

OR TI (health NEXT occupation*)

OR AB (health NEXT occupation*)

OR MH "Nursing"

OR TI nursing

OR AB nursing

OR MH "Dietetics"

OR TI dietetics

OR AB dietetics

OR TI dietitian*

OR AB dietitian*

OR TI dietician*

OR AB dietician*

OR MH "Pharmacology"

OR TI pharmacolog*

OR AB pharmacolog*

OR MH "Students, Health Occupations"

OR TI (health NEXT occupation NEXT student*)

OR AB (health NEXT occupation NEXT student*)

OR TI (health NEXT profession NEXT student*)

OR AB (health NEXT profession NEXT student*)

OR TI (healthcare NEXT student*)

OR AB (healthcare NEXT student*)

OR MH "Chiropractic"

OR TI chiropract*

OR AB chiropract*

OR MH "Students, Nursing"

OR TI (nurs* NEXT student*)

OR AB (nurs* NEXT student*)

OR MH "Occupational Therapy"

OR TI (occupational NEXT therap*)

OR AB (occupational NEXT therap*)

OR MH "Occupational Therapists"

OR TI (occupational NEXT therapist*)

OR AB (occupational NEXT therapist*)

OR TI (health NEXT occupation* NEXT student*)

OR AB (health NEXT occupation* NEXT student*)

OR MH "Speech Therapy"

OR TI (speech NEXT therap*)

OR AB (speech NEXT therap*)

OR MH "Nutritionists"

OR TI nutrition*

OR AB nutrition*

OR TI nutritionist*

OR AB nutritionist*

OR MH "Pharmacists"

OR TI pharmacist*

OR AB pharmacist*

OR MH "Students, Pharmacy"

OR TI (pharmacy NEXT student*)

OR AB (pharmacy NEXT student*)

OR MH "Students, Dental"

OR TI (dental NEXT student*)

OR AB (dental NEXT student*)

OR MH "Dentistry"

OR TI dentist*

OR AB dentist*

OR MH "Respiratory Therapy"

OR TI (respiratory NEXT therap*)

OR AB (respiratory NEXT therap*)

OR TI (respiratory NEXT care)

OR AB (respiratory NEXT care)

OR TI (respiratory NEXT therapy NEXT student*)

OR AB (respiratory NEXT therapy NEXT student*)

OR MH "Laboratory Personnel"

OR TI (medical NEXT technolog*)

OR AB (medical NEXT technolog*)

OR TI (clinical NEXT laboratory NEXT personnel)

OR AB (clinical NEXT laboratory NEXT personnel)

OR MH "Students, Medical"

OR TI (medical NEXT student*)

OR AB (medical NEXT student*)

OR MH "Health Personnel"

OR TI (health NEXT personnel)

OR AB (health NEXT personnel)

OR TI (health NEXT care NEXT personnel)

OR AB (health NEXT care NEXT personnel)

OR TI (allied NEXT health NEXT personnel)

OR AB (allied NEXT health NEXT personnel)

OR MH "Education, Medical"

OR TI (medical NEXT education)

OR AB (medical NEXT education)

OR TI (undergraduate NEXT student*)

OR AB (undergraduate NEXT student*)

OR MH "Education, Nursing, Research-Based"

OR TI (nursing NEXT education NEXT research)

OR AB (nursing NEXT education NEXT research)

OR MH "Education, Nursing, Baccalaureate"

OR TI (education NEXT nursing NEXT baccalaureate)

OR AB (education NEXT nursing NEXT baccalaureate)

OR MH "Education, Nursing, Diploma Programs"

OR TI (education NEXT nursing NEXT diploma NEXT programs)

OR AB (education NEXT nursing NEXT diploma NEXT programs)

OR MH "Education, Dental"

OR TI (education NEXT dental)

OR AB (education NEXT dental)

OR MH "Education, Pharmacy"

OR TI (education NEXT pharmacy)

OR AB (education NEXT pharmacy)

OR MH "Pharmacy Residencies"

OR TI (pharmacy NEXT residencies)

OR AB (pharmacy NEXT residencies)

OR TI (health NEXT student)

OR AB (health NEXT student)

OR TI (physical NEXT therap* NEXT student*)

OR AB (physical NEXT therap* NEXT student*)

OR TI (physiotherap* NEXT student*)

OR AB (physiotherap* NEXT student*)

)	12240

#2	(

MH "Clinical Clerkship"

OR TI (clinical NEXT clerkship)

OR AB (clinical NEXT clerkship)

OR MH "Mentorship"

OR TI mentoring

OR AB mentoring

OR MH "Preceptorship"

OR TI preceptorship

OR AB preceptorship

OR MH "Interns and Residents"

OR TI (internship NEXT nonmedical)

OR AB (internship NEXT nonmedical)

OR MH "Internship and Residency"

OR TI (internship NEXT residency)

OR AB (internship NEXT residency)

OR MH "Teaching Rounds"

OR TI (teaching NEXT round*)

OR AB (teaching NEXT round*)

OR TI internship*

OR AB internship*

OR TI (clinical NEXT placement*)

OR AB (clinical NEXT placement*)

OR TI (clinical NEXT education)

OR AB (clinical NEXT education)

OR TI (clinical NEXT training*)

OR AB (clinical NEXT training*)

OR TI practicum

OR AB practicum

OR TI (clinical NEXT rotation*)

OR AB (clinical NEXT rotation*)

OR TI (clinical NEXT experience*)

OR AB (clinical NEXT experience*)

OR TI (professional NEXT practice)

OR AB (professional NEXT practice)

OR TI (work-based NEXT learning)

OR AB (work-based NEXT learning)

OR TI (workplace NEXT learning)

OR AB (workplace NEXT learning)

OR TI (clinical NEXT supervision)

OR AB (clinical NEXT supervision)

OR TI (clinical NEXT teaching)

OR AB (clinical NEXT teaching)

OR TI fieldwork

OR AB fieldwork

OR TI (experiential NEXT learning)

OR AB (experiential NEXT learning)

OR TI (hands-on NEXT training)

OR AB (hands-on NEXT training)

OR TI apprenticeship*

OR AB apprenticeship*

OR TI (educational NEXT setting*)

OR AB (educational NEXT setting*)

OR TI (residency NEXT education)

OR AB (residency NEXT education)

OR TI (internship NEXT programs)

OR AB (internship NEXT programs)

OR TI (nursing NEXT preceptorship)

OR AB (nursing NEXT preceptorship)

OR TI (medical NEXT preceptorship)

OR AB (medical NEXT preceptorship)

)

AND

(

MH "Professional Autonomy"

OR TI "Professional Autonomy"

OR AB "Professional Autonomy"

OR TI (educational NEXT setting)

OR AB (educational NEXT setting)

OR TI (clinical NEXT learning NEXT environment)

OR AB (clinical NEXT learning NEXT environment)

OR TI (learning NEXT environment*)

OR AB (learning NEXT environment*)

OR TI (learning NEXT setting*)

OR AB (learning NEXT setting*)

OR TI quality

OR AB quality

)	641

#3	TI (focus NEXT group*)

OR AB (focus NEXT group*)

OR TI reliability

OR AB reliability

OR TI observation*

OR AB observation*

OR TI cohort

OR AB cohort

OR TI surve*

OR AB surve*

OR TI interview*

OR AB interview*

OR TI validation

OR AB validation	10329

#4	TI instrument*

OR AB instrument*

OR TI questionnaire*

OR AB questionnaire*

OR TI tool*

OR AB tool*

OR TI surve*

OR AB surve*

OR TI scale*

OR AB scale*

OR TI inventor*

OR AB inventor*

OR TI evaluat*

OR AB evaluat*

OR TI assess*

OR AB assess*

OR TI measur*

OR AB measur*

OR TI percept*

OR AB percept*

OR TI perspective*

OR AB perspective*

OR TI view*

OR AB view*	30908

#5	#1 AND #2 AND #3 AND #4	495

TOTAL: 8551 results
